# Meta-cancer phosphoproteomic analysis unveils association of Tau phosphosites with DNA damage response

**DOI:** 10.3389/fsysb.2026.1819794

**Published:** 2026-05-22

**Authors:** Tejaswini Rohinath Poojari, Leona Dcunha, Althaf Mahin, Athira Perunelly Gopalakrishnan, Mukhtar Ahmed, Pathiyil Sajini Sekhar, Samseera Ummar, Levin John, Prashant Kumar Modi, Sowmya Soman, Rajesh Raju, Akhina Palollathil

**Affiliations:** 1 Centre for Integrative Omics Data Science (CIODS), Yenepoya (Deemed to be University), Mangalore, Karnataka, India; 2 Department of Zoology, College of Science, King Saud University, Riyadh, Saudi Arabia; 3 Institute for Regeneration and Repair, University of Edinburgh, Edinburgh, Scotland, United Kingdom; 4 Centre for Systems Biology and Molecular Medicine (CSBMM), Yenepoya Research Centre, Yenepoya (Deemed to be University), Mangalore, Karnataka, India

**Keywords:** cancer, DNA damage response, DNA repair, FET, microtubule-associated proteins, phosphoproteomics, phosphosites, tau

## Abstract

**Introduction:**

The microtubule-associated protein Tau, a key regulator of microtubule stability, is associated with neuronal function and neurodegeneration. Emerging evidence indicates that Tau also participates in cancer-related signalling. However, a systematic understanding of Tau phosphorylation across cancers remains limited.

**Methods:**

We performed an integrative analysis of human cellular phosphoproteomics datasets to examine the Tau phosphorylation beyond its canonical neuronal roles.

**Results:**

The comprehensive analysis of global cellular phosphoproteomics datasets revealed that the Tau phosphosites, such as S519, S713, S717, and S721, were present across various experimental conditions. The co-regulation study revealed that a comprehensive network of phosphosites in other proteins (PsOPs) exhibited either positive or negative co-regulation with these Tau phosphosites, suggesting unique site-specific phosphorylation dynamics. Considering the specific functional characterisation of Tau phosphosites, we employed a global co-regulation analysis by assessing the phosphosite-specific functions of co-regulated PsOPs, including binary interactors, complex interactors, and upstream kinases. The functional roles that can be inferred from the co-regulation patterns show cancer-related processes, such as DNA damage response, DNA repair, cell motility, and cell growth. The co-regulation analysis showed mutual regulation among S713, S717, and S721, indicating coordinated phosphorylation within Tau, which was further substantiated by the co-occurrence analysis. Additionally, the upstream kinases identified for Tau, such as CDK12/13/14/16, GSK3B, RPS6KA3, and CSNK1E, are involved in DNA damage response and carcinogenesis. These results provide a phosphosite-centric view of Tau regulation and its signalling networks in cancer, underscoring the relevance of Tau phosphosites beyond neuronal biology.

## 1 Introduction

The microtubule-associated proteins (MAPs), including *MAP2*, *MAP4*, and Tau, play a critical role in binding actin and facilitating interactions between actin polymers and microtubules (Mohan and John, 2015). In addition, MAPs regulate diverse cellular functions by connecting microtubules to other cytoskeletal elements, organelles, and cellular membranes (Bodakuntla et al., 2019; Dehmelt and Halpain, 2005; Ruiz-Gabarre et al., 2022; Wang and Mandelkow, 2016). Among the MAPs, Tau is one of the most extensively studied proteins due to its involvement in several neurodegenerative disorders, collectively termed tauopathies, which are characterised by pathological Tau aggregation in neurons (Barbier et al., 2019). Tau was first identified in 1975 by Marc Kirschner as a protein that facilitates the polymerisation of tubulin. It is encoded by the *MAPT* gene, located on chromosome 17q21.31 (Ruiz-Gabarre et al., 2022). Alternative splicing of *MAPT* generates six Tau isoforms, ranging from 352 to 441 amino acids in length and has a molecular weight of 48–67 kDa (Hedna et al., 2022). In addition to neuronal expression, Tau have been detected in non-neuronal cells such as cardiomyocytes and epithelial cells in the lungs (Balczon et al., 2021; Betrie et al., 2017). At the subcellular level, Tau localizes predominantly to microtubules within the cytosol but has also been reported at the plasma membrane and within the nucleus and centrosome, depending on cell type and physiological context (Sotiropoulos et al., 2017). The primary structure of Tau consists of an N-terminal projection domain; it extends away from the microtubule, allowing it to interact with other cytoskeletal proteins or cytoplasmic organelles such as synaptic vesicles, annexins, or mitochondria (Hirokawa et al., 1988). The proline-rich region interacts with kinases and phosphatases to regulate Tau phosphorylation and, together with the microtubule-binding domain (MTBD), enhances microtubule assembly and stability. MTBD enables Tau to bind microtubules through interactions with tubulin, while the C-terminal domain facilitates microtubule association and induces conformational changes that help protect Tau from abnormal aggregation (Alquezar et al., 2020; Mietelska-Porowska et al., 2014; Wang and Mandelkow, 2016).

Tau is regulated by multiple PTMs (post-translational modifications), with phosphorylation playing a prominent role in modulating its cellular functions (Neddens et al., 2018). For instance, microtubule stabilization, spatial structure, and axonal transport regulation in neurons are all significantly influenced by site-specific phosphorylation of Tau (Cario and Berger, 2023). Specific kinases mediate phosphorylation of Tau at Ser404, Ser262, Ser235, and Ser202, which significantly impact microtubule dynamics (Trinczek et al., 1995). Changes in the Tau phosphorylation, either through hyperphosphorylation or aberrant kinase activity, can alter microtubule stability and intracellular transport, leading to neuronal disorders and disease (Alonso et al., 2018; Alquezar et al., 2020; Cho and Johnson, 2003; Turab Naqvi et al., 2020). In tauopathies, hyperphosphorylation of Tau impacts its functions, promotes aggregation, and leads to neuronal damage and disorders such as corticobasal degeneration (CBD), Alzheimer’s disease (AD), chronic traumatic encephalopathy (CTE) and frontotemporal dementia (FTD)(Gong and Iqbal, 2008; Zhang et al., 2024).

Beyond its established role in neuronal disorders, Tau has been associated with cancer, where it can influence p53-related signaling and promote cancer-associated processes such as inflammation, epithelial-mesenchymal transition, and cellular proliferation (Callari et al., 2023). Multiple studies have reported the association of Tau in cancers, including breast cancer (Matrone et al., 2010), hepatocellular carcinoma (Liu et al., 2025), bone cancer (Cidre-Aranaz et al., 2025), and clear cell renal cell carcinoma (Han et al., 2020). In addition, Rossi et al. (2018) documented that an autosomal dominant mutation in Tau can cause chromosome and genome instability, resulting in aneuploidy, a hallmark characteristic of cancer cells (Rossi et al., 2018). Emerging evidence indicates that Tau phosphorylation may contribute to its functional roles in cancer. Hyperphosphorylated Tau has been observed in multiple malignancies, including prostate cancer, neuroblastoma, ovarian carcinoma, and colorectal cancer, where it is associated with altered microtubule dynamics, cell-cycle dysregulation, and therapy resistance (Clementi et al., 2023; Flores-Rodriguez et al., 2019; Souter and Lee, 2009; Yang et al., 2017). In cancer cells, phosphorylation of Tau at Thr231 has been shown to occur in a cell-cycle-dependent manner, suggesting a potential role for Tau in mitotic regulation (Clementi et al., 2023). Additionally, phosphorylation of Tau at Ser396 and Thr181 has been detected in chemotherapy-resistant ovarian tumours, further relating the aberrant Tau phosphorylation to aggressive cancer phenotypes (Barbolina, 2022). While individual Tau phosphosites have been investigated in specific tumour models, a systematic understanding of Tau phosphorylation across diverse cancer types remains limited.

To address this knowledge gap, we systematically integrated large-scale cellular phosphoproteomics datasets to specify the phosphorylation landscape of Tau across diverse biological and experimental contexts. This analysis enabled us to uncover patterns of Tau phosphosites that frequently co-occur or are co-regulated, revealing Tau-associated phosphosignaling in carcinogenesis. Collectively, this work establishes a future investigation into phosphosite-specific functions of Tau and the exploration of Tau-centred signalling networks as potential therapeutic targets in tumour and other diseases.

## 2 Methodology

### 2.1 Integrative analysis of global phosphoproteomics datasets of Tau

The global cellular phosphoproteomics datasets analysed in this study were curated and compiled as described previously (Palollathil et al., 2026; Priyanka et al., 2024; Raghu et al., 2025; Sujina et al., 2025). This process involved a comprehensive literature survey conducted via PubMed, employing the MeSH terms, such as “phosphoproteomics” OR “phosphoproteome”, while excluding entries related to “plants” and “review articles”. Subsequently, human cellular phosphoproteomics datasets were retrieved, specifically focusing on Class-1 phosphosites characterised by a localisation probability of at least 75% or an A-score of 13 or higher.

The datasets were then divided into two categories, the first category as qualitative profiles, where test and control samples were looked at separately to make a complete list of all the phosphopeptides that were found. The second category includes quantitative differential, where phosphosite abundance in test conditions was quantitatively compared with their corresponding controls, facilitating the identification of differentially regulated phosphopeptides. For differential profile datasets, we further defined significance as *p-value* less than 0.05 and fold change values of greater than or equal to 1.3 as upregulated proteins and less than or equal to 0.76 as downregulated proteins. Furthermore, proteins were standardised by mapping them to their official gene symbols (as defined by the HGNC, Human Gene Nomenclature Committee), along with their UniProt accession number, to ensure consistency (Lalu et al., 2025).

### 2.2 Characterisation of predominant Tau phosphosites within phosphoproteomics datasets

The detection frequency of each class-1 Tau phosphosites was determined by calculating how often it appeared in qualitative profiles and quantitative differential datasets and then ranking these counts. Tau phosphosites showed a broad range of detection frequencies across these datasets, with some phosphosites rarely observed and others consistently detected. Phosphosites that appeared most often across various profiling and differential phosphoproteomics datasets were considered as the predominant Tau phosphosites (Palollathil et al., 2026; Priyanka et al., 2024; Raghu et al., 2025; Sujina et al., 2025).

### 2.3 Protein phosphosites that are co-regulated with predominant phosphosites of the Tau

To identify PsOPs that exhibit positive or negative co-regulation with each predominant Tau site, the differential abundance datasets were classified into four groups. When a Tau phosphosite was upregulated, PsOPs with increased and decreased abundance were categorized as UtauUothers and UtauDothers, respectively. Conversely, when a Tau phosphosite was downregulated, PsOPs with decreased and increased abundance were classified as DtauDothers and DtauUothers, respectively. These four patterns were combined into two broader categories, UUDD (UtauUothers + DtauDothers), reflecting positive co-regulation, and UDDU (UtauDothers + DtauUothers), reflecting negative co-regulation. PsOPs were ranked by the frequency with which they exhibited these patterns across datasets, and only those supported by multiple observations were retained to avoid false associations. This approach was applied to all predominant Tau phosphosites (Fathima et al., 2025).

### 2.4 Co-occurrence analysis

Phosphorylation changes were classified as upregulated (U) or downregulated (D), and the frequencies of the four possible patterns U(T1)U(T2), U(T1)D(T2), D(T1)D(T2), and D(T1)U(T2) were quantified for each phosphosite pair (T1, T2). Positive co-regulation was estimated using the ratio Σ[nU(T1)U(T2) + nD(T1)D(T2)]/Σ[nU(T1)D(T2) + nD(T1)U(T2)], whereas negative co-regulation was defined by the inverse ratio. Higher ratio values indicate stronger concordant or discordant regulation between phosphosite pairs.

This approach enables quantification of phosphosite-level dependency and facilitates the identification of tightly co-regulated Tau phosphorylation events across datasets (Parplys et al., 2015; Kammarambath et al., 2026).

### 2.5 Filtering phosphosites to prevent biases and over-representation in datasets

To minimize potential sources of bias, such as the over-representation of phosphosites originating from one or two studies or from large multi-temporal datasets generated under a single stimulation type, an additional layer of data filtering was applied to define high-confidence co-regulated phosphosites. In this procedure, PsOPs with a FET *p*-value less than 0.05 were further evaluated based on the ratio ∑(nUU + nDD)/∑(nUD + nDU) for positive co-regulation and ∑(nUD + nDU)/∑(nUU + nDD) for negative co-regulation (Palollathil et al., 2026; Priyanka et al., 2024; Raghu et al., 2025; Sujina et al., 2025).

PsOPs that met a frequency threshold representing at least 10% of total detections of the specific predominant Tau phosphosites were retained for further consideration. To reduce bias introduced by datasets derived from similar biological contexts or repeated experimental conditions, an additional requirement was imposed: a given co-regulation pattern (positive or negative) had to be supported by evidence from at least three different experimental conditions and three distinct studies. Collectively, only those phosphosites from other proteins that fulfilled these criteria were designated as high-confidence co-regulation partners of predominant Tau phosphosites (Palollathil et al., 2026; Priyanka et al., 2024; Raghu et al., 2025; Sujina et al., 2025).

Fisher’s extract test (FET) was performed using contingency tables constructed to evaluate the association between individual Tau phosphosites and corresponding PsOPs.

Fisher’s exact test (FET):

$$\sum p=\frac{\left(a+b\right)!\left(c+d\right)!\left(a+c\right)!\left(b+d\right)!}{n!}\;\sum_{i}\frac{1}{a_{i}!b_{i}!c_{i}!d_{i}!}$$

Within this framework, “a” denotes conditions in which neither phosphosite was detected, “b” represents detection of only one of the two sites (irrespective of direction of regulation), “c” corresponds to negative regulation between the paired sites, and “d” reflects positive regulation. For each phosphosite pair, Fisher’s exact test (FET) was applied to the corresponding contingency table to evaluate the statistical significance of co-regulation. Pairs with an FET *p*-value < 0.05 in either the positive (UUDD) or negative (UDDU) category were retained for downstream analysis (Sujina et al., 2025).

### 2.6 Compilation of Tau-interacting proteins

Experimentally validated protein-protein interactors and associated proteins of Tau were retrieved from databases such as BioGRID (Oughtred et al., 2019), HPRD (Keshava Prasad et al., 2009), ConsensusPathDb version 35 (Kamburov and Herwig, 2022), and BIND (Bader et al., 2003).

### 2.7 Extraction of upstream kinases of the Tau protein

To explore the upstream kinases that phosphorylate Tau protein, we curated data from databases, such as PhosphoSitePlus (downloaded on May 22, 2023) (Hornbeck et al., 2015), Phospho.ELM 9.0 (downloaded on May 24, 2023) (Dinkel et al., 2011), and RegPhos 2.0 (downloaded on May 24, 2023) (Huang et al., 2014). In addition to these, upstream kinases specific to the Tau protein were predicted using numerous *in silico* prediction tools such as NetworKIN (predicted on January 04, 2023) (Linding et al., 2008), AKID (predicted on May 24, 2023) (Parca et al., 2019), and iKiP-DB (Mari et al., 2022), which were also collected to analyze upstream kinases. Furthermore, kinases targeting Tau phosphosites identified through synthetic peptide screening by Johnson et al. (2023), applying a 90th percentile cutoff, were also incorporated into the analysis (Johnson et al., 2023).

### 2.8 Data visualization

Lollipop plots were generated using the trackViewer, a R/Bioconductor package (Ou and Zhu, 2019). The distribution of phosphosites across profiling and differential datasets were illustrated with the Python(v3.13.0) (Han and Kwak, 2023) libraries, Matplotlib(v3.10.0) and Pandas(v2.3.3). Circular dendrograms displaying co-regulation patterns with interactors, upstream kinases, and substrates were generated using RAWGraphs 2.0 (https://app.rawgraphs.io/), Cytoscape (v3.10.4), Adobe Illustrator (2020), and BioRender (2023).

### 2.9 Protein level expression of Tau

The protein level expression of Tau in human tissues and organs was assessed by utilizing the protein abundance data from the Human Protein Atlas (https://www.proteinatlas.org/).

## 3 Results

### 3.1 Global phosphoproteomic analysis of Tau phosphorylation and identification of its predominant phosphosites

**FIGURE 1:**
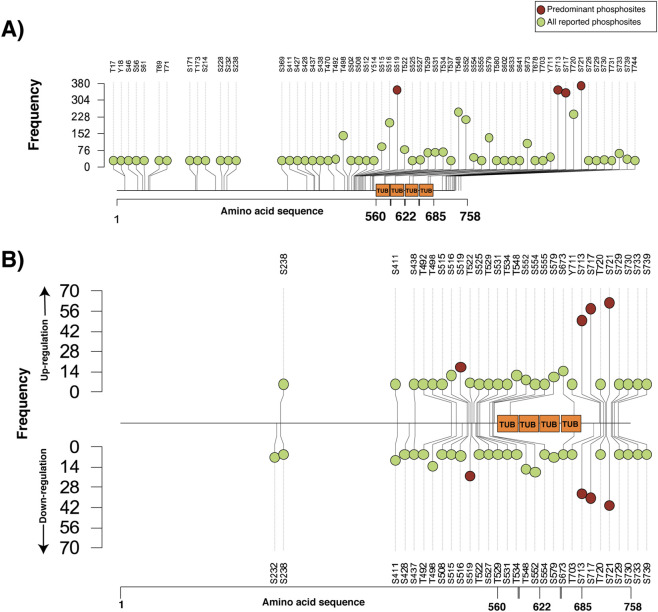
Lollipop plot depicting phosphosites of Tau observed from human cellular profiling and the differential phosphoproteomics datasets. The X-axis represents the amino acid sequence length, and the Y-axis represents the number of datasets in which specific Class-1 phosphosites were detected. **(A)** Phosphosites of Tau identified in human cellular profiling datasets. **(B)** Phosphosites of Tau identified in human cellular differential datasets.

Phosphoproteomics has become a powerful approach to mapping phosphorylation-driven signalling networks and elucidating how protein modifications regulate cellular function (Palollathil et al., 2021; Rex et al., 2022). Tau phosphorylation has been explored in recent studies, yet its roles in cancer-related cellular contexts remain insufficiently characterized. To address this limitation, this study analysed Tau phosphorylation patterns to understand their potential functions and significance in diverse cancers. A total of 3825 publicly available mass spectrometry-based phosphoproteomics studies were systematically analysed to identify predominantly and poorly detected Tau phosphosites. This resulted in the retrieval of 679 phosphoproteomic profiling datasets and 222 differential abundance datasets containing Tau phosphosite information (Supplementary Tables S1a,S1b). We identified several Class-I phosphosites that were frequently observed across multiple datasets derived from different experimental conditions. These phosphosites were further evaluated and ranked based on the detection frequency across various experimental conditions. Within differential datasets, Tau phosphosites such as S721, S717, S713, and S519 emerged as the top frequently identified phosphosites, with frequencies of 105, 96, 85, and 41, respectively. Given their consistently higher frequencies compared to other reported phosphosites in Tau, we designated S721, S717, S713, and S519 as the predominant phosphosites of Tau, shown in Figure 1.

### 3.2 Co-occurrence analysis of Tau phosphosites

**FIGURE 2:**
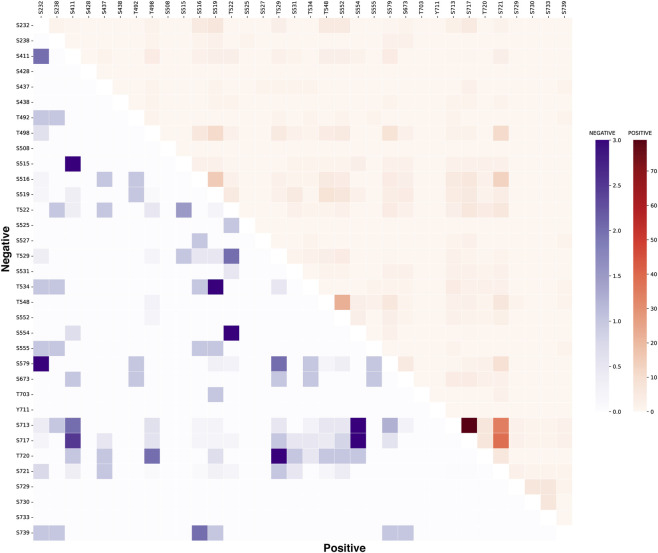
Heatmap showing the co-occurrence patterns of Tau phosphosites across differential phosphoproteomics datasets. Colour intensity reflects the frequency of positive and negative co-occurrence between phosphosites.

The analysis of co-occurring phosphosites of a protein provides insight into shared regulatory mechanisms and potential functional relevance (Li et al., 2017). To investigate this further in the context of Tau, we analysed the co-occurrence of other phosphosites within Tau alongside the predominant phosphosites S519, S713, S717, and S721. Interestingly, we observed strong positive co-occurrence among the predominant Tau phosphosites, including S713, S717, S719, and S721. In particular, the S713 exhibited a strong co-occurrence with other predominant phosphosites such as S717 and S721. The co-occurrence of S713 with S717 was detected in 79 positively co-regulated datasets, with only one dataset showing negative co-regulation, indicating a strong bias toward coordinated phosphorylation. Likewise, S713 and S721 co-occurred in 69 positively co-regulated datasets, compared with only two negatively co-regulated instances. S717 also exhibited strong positive co-occurrence with S721, supported by 77 positive and only two negative co-regulation datasets. In addition, S719 displayed predominantly positive co-occurrence with S721, with evidence from 18 positively co-regulated datasets and only five negatively co-regulated datasets. In contrast, non-predominant phosphosites such as S552 and T548 showed coordinated phosphorylation, with co-occurrence observed only in 22 positively co-regulation datasets (Figure 2; Supplementary Table S1c). Collectively, the coordinated phosphorylation of predominant Tau phosphosites suggests shared or interrelated functional roles.

### 3.3 Analysis of protein phosphosites that are co-regulated with predominant Tau phosphosites

To assess the patterns of co-regulation among PsOPs and Tau predominant phosphosites, the datasets were initially categorised according to the differential expression, distinguishing upregulated and downregulated phosphosites. Based on these, co-expression relationships were defined as UUDD for positive co-regulation and UDDU for negative co-regulation. To evaluate the likelihood and statistical confidence of these associations, a contingency table was constructed and analysed using a one-sided FET. The analysis encompassed 222 differential datasets in which Tau phosphosites were identified. For each phosphosite pair (a predominant site and its corresponding phosphosite of interest), the contingency table summarises datasets in which (i) neither site was differentially regulated, (ii) only one site showed regulation. (iii) both phosphosites exhibited opposing regulation, or (iv) both showed coordinated up or downregulation. The FET *p*-value cutoff was set to less than 0.05, and phosphosite pairs with values below this threshold were considered statistically significant for co-regulation. To determine high-confidence co-regulation patterns among the phosphosites, additional filtering criteria were applied for Tau-associated phosphosites. The filtering criteria were detailed in the methodology.

In the analysis, the Tau exhibited a notable co-regulation with PsOPs, indicating a distinct regulatory pattern across its phosphorylation network. Specifically, the predominant phosphosite S519 exhibited 838 positively and 30 negatively co-regulated phosphosites, reflecting a strong bias toward positive regulation. Likewise, the predominant phosphosites S717, S713, and S721 were positively co-regulated with 60, 76, and 127 phosphosites on other proteins, respectively, while they were negatively co-regulated with 9, 11, and 2 phosphosites. This distribution highlights the differential phosphorylation dynamics within Tau, suggesting certain phosphosites, particularly S519, serve as a major phosphorylated site across various experimental conditions in Tau protein (Supplementary Tables S2a,S2b).

**FIGURE 3:**
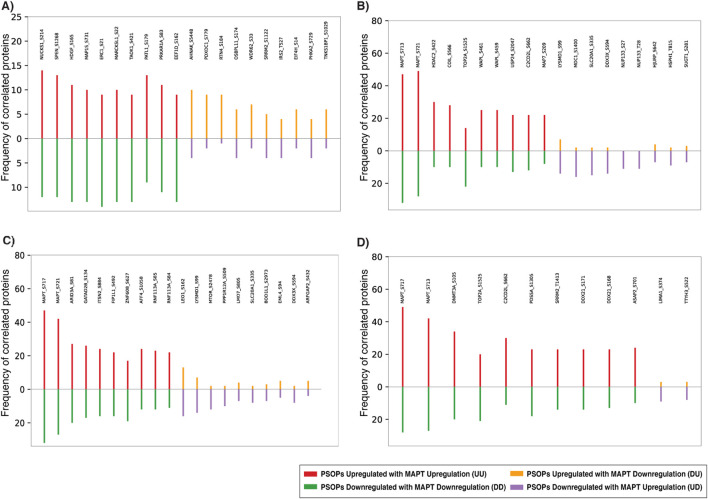
Bar graphs showing the top positively and negatively co-regulated proteins associated with the major Tau (MAPT) phosphosites. The top 10 proteins whose phosphosites display the strongest positive (up-up or down-down) or negative (up-down or down-up) co-regulation with each predominant Tau phosphosite, such as **(A)** S519, **(B)** S717, **(C)** S713, and **(D)** S721, across the dataset.

Among the positively co-regulated phosphosites associated with Tau S519, NUCKS1 (S214) showed the highest frequency of 26, whereas AHNAK (S5448) was the strongly negatively co-regulated protein for this site, with a frequency of 14 (Figure 3A). Interestingly, Tau S713 exhibited its strongest positive co-regulation with another Tau phosphosite, S717, showing regulation within the protein (Figure 3B). Similarly, 79 datasets showed the positive co-regulation between MAPT (Tau) S717 and Tau S713, making them the most frequently detected co-regulated phosphosites in this analysis. This further supports the presence of closely related phosphosites within Tau (Figure 3C). Tau S721 also exhibits strong positive co-regulation with Tau S717, with a frequency of 77 (Figure 3D). In addition to these positive associations, Tau S713, S717, and S721 showed strong negative co-regulation with LEO1 (S162), LYSMD1 (S99), and LIMA1 (S374), with frequencies of 29, 21, and 12, respectively. The top 10 positively and negatively co-regulated PsOPs of Tau predominant phosphosites are represented in Figures 3A–D.

**FIGURE 4:**
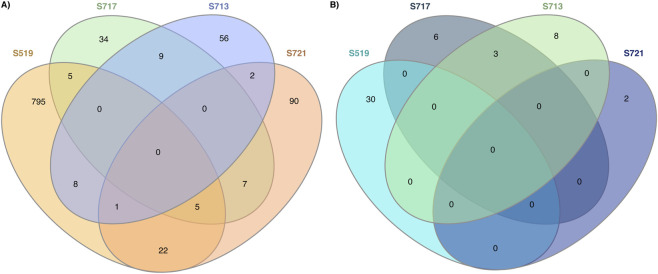
Comparative analysis of PsOPs associated with each predominant phosphorylation site of Tau. Venn diagrams showing the overlap of **(A)** positively co-regulated PsOPs and **(B)** negatively co-regulated PsOPs shared among the predominant phosphorylation sites.

To examine the shared PsOPs among the predominant phosphosites, we performed a comparative analysis using InteractiVenn. Among the positively regulated PsOPs of predominant phosphosites, we found 22 common PsOPs between S519 and S721. Similarly, we noted two common PsOPs between S713 and S721, and eight common PsOPs between S519 and S713. Additionally, seven common PsOPs were identified among predominant phosphosites S717 and S721, as well as S713 and S717. In the negatively regulated PsOPs of predominant phosphosites, three common PsOPs were found among S717 and S713 (Figures 4A,B).

### 3.4 Functional enrichment of proteins co-regulated with Tau predominant phosphosites

**FIGURE 5:**
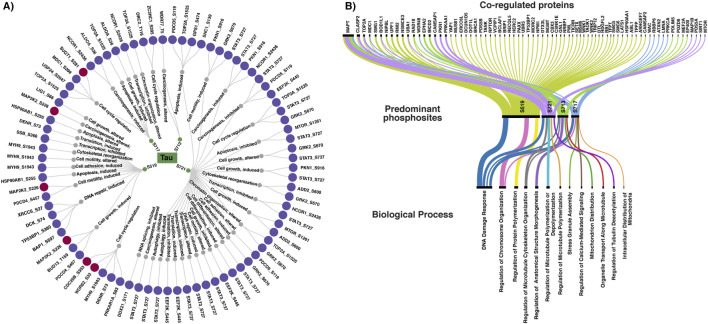
Biological processes associated with phosphosites co-regulated with Tau predominant phosphosites. **(A)** Biological functions are regulated by specific phosphosites of highly co-regulated proteins. **(B)** Enriched biological processes of co-regulated proteins identified using Enrichr.

The biological functions regulated by Tau phosphosites remain poorly established, limiting our understanding of phosphosite-specific Tau-dependent regulatory mechanisms. Given that co-regulated phosphosites in proteins often participate in related cellular processes and pathways (Kustatscher et al., 2019; Li et al., 2017). To infer potential site-specific functions of the predominant Tau phosphosite, the biological roles of its PsOPs were examined. The phosphosite-specific biological functions of positively and negatively co-regulated PsOPs were retrieved from the PhosphoSitePlus database. Overall, the phosphosites co-regulated with the predominant Tau phosphosites S519, S713, S717, and S721 were primarily associated with key cellular processes, including cell cycle regulation (TOP2A_S1525, STAT3_S727, PRKAR1A_S83), carcinogenesis (MYH9_S1943, EEF2K_S445, PKN1_S916), cell growth (GRK2_S670, PDCD4_S457, BUD13_T159, PKN1_S916), cell motility (GRK2_S670, PKN1_S916, MYH9_S1943, STAT3_S727), and apoptosis (HSP90AB1_S255, PDCD5_S119, TOP2A_S1525, MTOR_S1261). The complete data regarding the PsOPs and their site-specific functional roles are given in Supplementary Table S3. These observations indicate that Tau-related phosphorylation events may contribute to key regulatory processes that support tumour initiation and progression. Notably, the phosphoproteins co-regulated with S519 (such as TP53BP1_S380, DCK_S74, XRCC6_S27, BAP1_S597), and S721 (such as STAT3_S727) showed phosphosite-specific functions in DNA repair (Figure 5A).

Furthermore, functional enrichment analysis using the Enrichr platform was performed to explore the biological processes associated with these PsOPs at the protein level. Consistent with the phosphosite-specific functions described above, the enrichment results also showed a strong enrichment of DNA damage response among proteins co-regulated with each predominant Tau phosphosites. In addition to this, these co-regulated proteins were also associated with biological processes such as regulation of protein polymerisation, regulation of microtubule cytoskeleton organisation, and stress granule assembly, as shown in Figure 5B.

### 3.5 Tau in cancer biology

**FIGURE 6:**
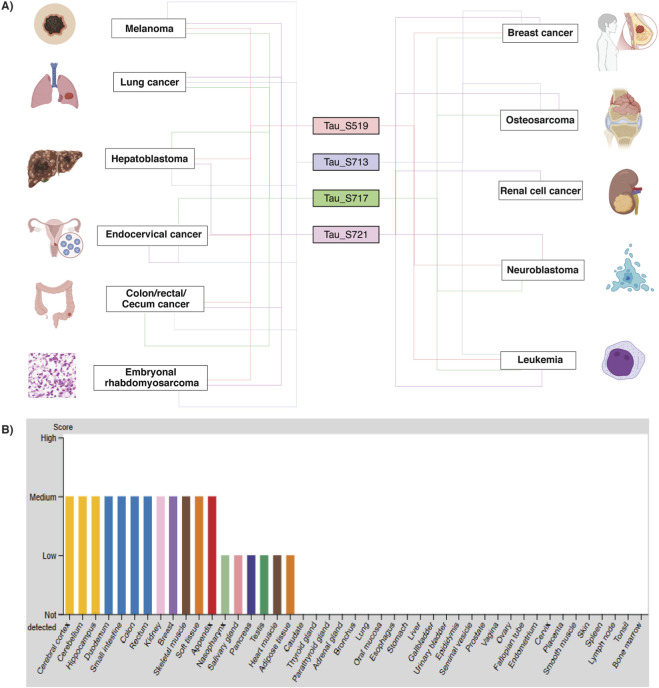
Tau phosphorylation across cancer types and protein expression patterns in human organs. **(A)** Graphical representation showing the differential expression pattern of four predominant Tau phosphosites (S519, S713, S717, and S721) across diverse human cancers. **(B)** Tau exhibits distinct organ-specific expression patterns at protein levels. Protein expression levels are presented across 44 tissues using knowledge-based annotation, with colour coding reflecting tissue group classifications that share common functional characteristics.

Since the phosphoproteomic datasets were derived from diverse cancer cell lines, we further examined cancer-specific phosphorylation patterns of Tau predominant phosphosites. Out of 222 differential datasets, 198 datasets were cancer-related. Among the cancer-related datasets, Tau predominant phosphosites, such as S519, S713, S717, and S721, were reported in 14, 63, 6, and 10 datasets, respectively. This analysis revealed that Tau predominant phosphosites were frequently detected in a hyperphosphorylated state across multiple cancer types. The differential regulation of Tau predominant phosphosites, such as S519, S713, S717 and S721, were commonly observed in breast adenocarcinoma, cecum adenocarcinoma, colon adenocarcinoma, endocervical adenocarcinoma, hepatoblastoma, leukaemia, and melanoma (Figure 6A). In particular, upregulation of all four predominant phosphosites was observed in breast adenocarcinoma, leukaemia, and cecum adenocarcinoma, whereas three phosphosites (S721, S717, and S713) were upregulated in lung adenocarcinoma, melanoma, and osteosarcoma. Conversely, the downregulation of all four predominant phosphosites was identified in melanoma, leukaemia, hepatoblastoma, and colon adenocarcinoma. The detailed information on Tau predominant phosphosites expression pattern in cancer types, along with the corresponding cell lines used, is provided in Supplementary Table S4.

Further, we also analysed the organ-specific expression pattern of Tau using the Human Protein Atlas Database (HPA). The results showed elevated expression of Tau in the cerebral cortex, hippocampus, colon, rectum, breast, kidney, and pancreas, among others (Figure 6B). Consistent with these findings, our differential phosphoproteomic data derived from various cancer cells also showed differential phosphorylation of Tau protein expression in breast cancer and colon cancer. Together, these findings indicate that Tau exhibits tissue-spanning expression and dynamic phosphorylation patterns in multiple malignancies, supporting its potential role in cancer-associated regulatory mechanisms beyond the nervous system.

### 3.6 Tau-centric binary and complex protein interactor

**FIGURE 7:**
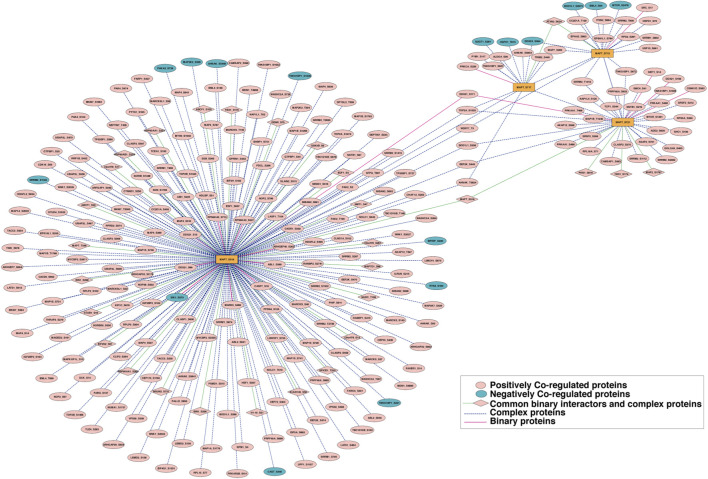
Network map showing the binary interactors and complex-associated proteins of Tau. Positively and negatively co-regulated interactors are highlighted according to the colour scheme.

The currently known binary and complex protein interactors of Tau were compiled from different resources, such as HPRD, BioGRID, BIND, RegPhos 2.0, CORUM and ConsensusPathDb to analyse site-specific phosphorylation dynamics associated with Tau. Our analysis identified positive and negative co-regulated binary and complex protein interactions associated with the predominant Tau phosphosites. Among the positively co-regulated proteins, the predominant site S519 was related to 35 binary interactors and 205 complex-associated proteins. Tau S717 was related to four binary interactors and 11 complex proteins, whereas S713 showed four binary interactors and 16 complex-associated proteins. For S721, 10 binary interactors and 35 complex proteins were identified. Among the negative PsOPs, S519 was related to one binary interactor and nine complex-associated proteins. The predominant phosphosites S717 and S713 were each related to three and four complex proteins, respectively. Moreover, our analysis revealed that 32 proteins were shared between the binary interactors and complex proteins in the compiled Tau datasets (Figure 7; Supplementary Tables S5a,S5b).

### 3.7 Upstream kinases of Tau

**FIGURE 8:**
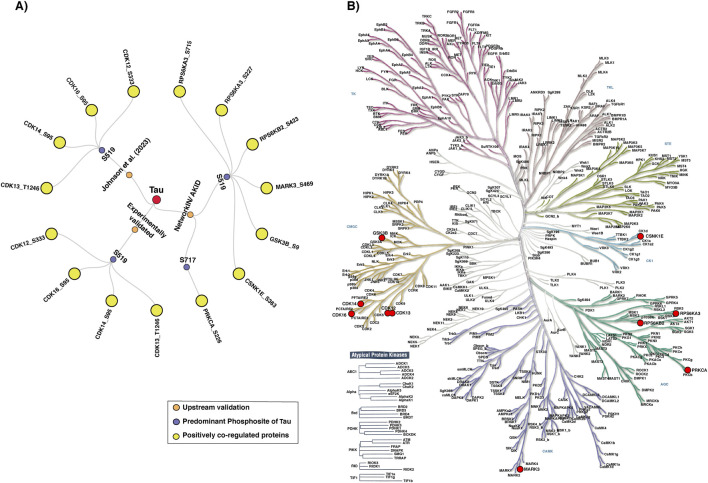
Upstream kinase network and KinMap classification of Tau regulatory kinases. **(A)** The network diagram illustrates the upstream kinases of Tau and their associated phosphosites. **(B)** KinMap showing the phylogenetic distribution of Tau-associated upstream kinases.

From the compiled data, a total of 10 unique upstream kinases were identified as potential regulators of Tau phosphorylation. Among these, four kinases, such as CDK12 (S333), CDK13 (T1246), CDK14 (S95), and CDK16 (S95), have been experimentally validated in previous studies to phosphorylate Tau at the S519 phosphosite, and this kinase-substrate association was further supported by kinase prediction analyses reported by Johnson et al. (2023) (Johnson et al., 2023). In addition, five kinases, such as RPS6KA3 (S227/S715), RPS6KB2 (S423), MARK3 (S469), GSK3B (S9), and CSNK1E (S363), were computationally predicted to phosphorylate Tau at the S519 phosphosite, whereas PRKCA (S226) was predicted to phosphorylate Tau at the S717 phosphosite (Figure 8A; Supplementary Table S6). These predictions were derived using established kinase-substrate prediction tools, such as AKID and NetworkIN. This integrated dataset combines both experimental evidence and computational predictions, providing a broader understanding of the potential regulatory network of Tau phosphorylation.

The mapping of upstream kinases of Tau using the KinMap tool revealed that these kinases were distributed across multiple major kinase families, including, Glycogen Synthase Kinase (GSK), Casein Kinase (CK1), Cyclin-Dependent Kinase (CDK), Calcium/Calmodulin-Dependent Protein Kinase-Like family (CAMKL), Protein Kinase C (PKC) and Ribosomal S6 Kinase (RSK) families (Figure 8B). A prominent cluster was mapped to the CDK family, including CDK12, CDK13, CDK14, and CDK16, indicating a strong contribution of cell cycle-associated regulatory kinases to Tau phosphorylation. Two kinases, such as RPS6KB2 and RPS6KA3, were grouped within the RSK family. The remaining kinases, such as CSNK1E, MARK3, PRKCA, and GSK3B, were mapped to the CK1, CAMKL, PKC, and GSK families, respectively.

## 4 Discussion

Tau is a microtubule-associated protein mainly localised in axons, where it supports microtubule stabilisation and assembly, promotes tubulin polymerisation, and regulates axonal transport (Hong et al., 2025). Tau is tightly regulated by coordinated activities of kinases and phosphatases, which control its phosphorylation status. This dynamic regulation is essential for maintaining normal neuronal structure and function (Yu et al., 2009). Abnormal phosphorylation of Tau alters its conformation, compromising its ability to bind and stabilise microtubules and thereby contributing to cytoskeletal disruption and neuronal dysfunction. In addition to phosphorylation, Tau is also regulated by other PTMs, including acetylation, methylation, glycosylation, and ubiquitination; however, their specific roles in disease onset and progression remain poorly understood (Alquezar et al., 2020; Kalyaanamoorthy et al., 2024). Apart from its canonical neuronal role, Tau expression has been reported in multiple cancers, including breast cancer, gastric cancer, and lung cancer, where it correlates with tumour responses to microtubule-targeting agents and other anticancer therapies (Papin and Paganetti, 2020). In tumours of neural origin, such as neuroblastoma and glioma, Tau expression is associated with improved patient survival (Callari et al., 2023; Zaman et al., 2018).

Among the top co-regulated PsOPs identified, NUCKS1_S214 and SPEN_S1268 were the top positively co-regulated phosphosites with Tau predominant site S519. NUCKS1 is a chromatin-associated factor with a well-established role in the homologous recombination and DNA damage response, key processes essential for maintaining genomic integrity and suppressing tumour development (Parplys et al., 2015). The co-regulation of Tau with NUCKS1 protein supports a functional relation between Tau and DNA repair pathways. SPEN is a transcriptional corepressor, and mutations in SPEN have been reported in the ERα-expressing T47D breast cancer cell line. It acts as a tumour suppressor that regulates cell proliferation, survival, and tumour progression (Legare et al., 2015). AHNAK_S5448 and PDXDC1_S779 were the negatively co-regulated PsOPs of S519. AHNAK, originally identified as a neuroblast differentiation-associated protein, is downregulated in colorectal, ovarian, and melanoma cancers, where its loss has been associated with enhanced tumour growth and metastasis through dysregulation of Wnt/β-catenin signalling and cadherin-1 expression (Cai et al., 2021; Cho et al., 2020; Sheppard et al., 2016). PDXDC1, which shows a significant association with glioblastoma, further reinforces the involvement of Tau-associated networks in aggressive tumour biology (Cannon-Albright et al., 2021). For the predominant phosphosite Tau_S717, HDAC2_S422 emerged as the most strongly positively co-regulated phosphoprotein. HDAC2 is a histone deacetylase whose aberrant expression contributes to cancer progression, and its targeted suppression using siRNA has been shown to induce tumour cell death (Marchion et al., 2009). MDC1_S1400 was identified as a highly negatively co-regulated phosphosite associated with Tau_S717. MDC1 is an early-response protein in the DNA damage response that facilitates the recruitment of additional DNA damage response factors to sites of DNA double-strand breaks, including BRCA1, 53BP1, and the MRN complex (Lou et al., 2003; Mochan et al., 2003; Ruff et al., 2020). Frequent downregulation of MDC1 has been reported in breast, laryngeal, and endometrial carcinomas, which hinders DNA damage response signaling and impairs efficient DNA repair, thereby promoting genomic instability, mutagenesis, and tumorigenesis (Gou et al., 2015; Merentitis et al., 2019; Zou et al., 2015). ARID3A_S81 is a positively co-regulated phosphosite of the Tau S713 predominant phosphosite. ARID3A is a transcription factor, known to be upregulated by p53 under DNA damage induced by UV radiation or doxorubicin treatment (Ma et al., 2003). Further studies revealed that ARID3A interacts with the DNA-binding domain of TP53, suppressing its transcriptional activation of tumour suppressor genes and thereby promoting tumour growth (Cai et al., 2025; Lestari et al., 2012). LEO1_S162 is a negatively co-regulated phosphosite of Tau S713. LEO1, a component of the RNA polymerase II-associated factor (PAF) complex, plays a critical role in the progression of acute myelogenous leukaemia by inducing the expression of oncogenes, such as SOX2 and SOX4 (Chong et al., 2014). LEO1 interacts with Cockayne syndrome protein B (CSB) and is recruited to chromatin in response to transcription-blocking DNA lesions induced by UV irradiation and cisplatin, and its depletion sensitizes cells to these genotoxic stresses, leading to increased cell death (Tiwari et al., 2021). TOP2A_S1525 was identified as a positively co-regulated phosphosite associated with the predominant Tau phosphosite S721. TOP2A regulates chromosome condensation, chromatid segregation, and alleviates torsional stress generated during DNA transcription and replication. Its upregulation contributes to malignant transformation (Chen et al., 2015; Liu et al., 2024). LIMA1_S374 is a negatively co-regulated phosphosite of S721 is a cytoskeleton-associated protein involved in gene regulation, cell cycle control, and cell migration. Its downregulation disrupts cytoskeletal dynamics, promoting tumour progression through enhanced invasion and migration, whereas its overexpression has been shown to reverse malignant phenotypes (Wang et al., 2023b). Collectively, the co-regulation of Tau predominant phosphosites with multiple DNA damage response and repair-associated proteins highlights a potential role for Tau in modulating DNA damage signaling and genomic stability in cancer contexts.

Further, the phosphosite-specific functions of the co-regulated PsOPs were evaluated using the PhosphoSitePlus database, which revealed the involvement of these sites in cell growth, cell motility, inhibition of apoptosis, and DNA repair. These findings suggest that Tau may contribute to key biological processes associated with cancer development and progression (Stuelten et al., 2018). Interestingly, the enrichment of DNA damage response, regulation of microtubule cytoskeleton organisation, and stress granule assembly at the protein level is particularly relevant in the context of Tau biology. As a microtubule-associated protein, Tau is a key regulator of microtubule polymerisation and stability, thereby regulating intracellular transport (Barbier et al., 2019). Recent studies indicate that, in response to DNA damage, Tau facilitates the nuclear translocation of the DNA repair mediator 53BP1, thereby causing resistance to conventional anti-cancer treatment (Rico et al., 2022). In addition, Tau has been implicated in the dynamics of stress granule assembly, where it may influence RNA metabolism and translational control (Cruz et al., 2019; Koren et al., 2020).

Furthermore, upstream kinase analysis identified CDK12, CDK13, CDK14, and CDK16 as kinases that are experimentally validated to phosphorylate Tau at the S519 phosphosite. Among these, CDK12 and CDK13 are expressed in neurons, where they regulate the expression of the downstream effector kinase CDK5, which is essential for axonal elongation (Chen et al., 2014). Notably, CDK5 has been reported to phosphorylate Tau at 45 distinct sites in the Alzheimer’s disease brain (Kimura et al., 2014), supporting a functional kinase cascade that modulates Tau phosphorylation. In addition, CDK12 plays a critical role in maintaining genomic stability by regulating the transcription of DNA damage response genes and is required for the proliferation and migration of neural progenitor cells (Chen et al., 2017), further highlighting Tau-associated phosphorylation networks in DNA repair and neurodevelopmental processes. CDK14 and CDK16 were reported to be involved in various cancers by regulating cell proliferation, apoptosis, and DNA damage response (Chen et al., 2019; Wang et al., 2023a). GSK3B_S9 was identified as the predicted upstream kinase of the predominant S519 phosphosite. GSK3B has been reported in carcinogenesis (Liu et al., 2023; Zhang et al., 2023) and is also known to hyperphosphorylate Tau at specific sites, resulting in neuronal disorder (Hooper et al., 2008). Moreover, the predicted upstream kinases of Tau, such as RPS6KA3, RPS6KB2, CSNK1E, and PRKCA, are reported to be involved in DNA damage pathways, immune infiltration in cancer, cell migration, invasion, and drug resistance (Chi et al., 2025; Lim et al., 2013; Ma et al., 2024).

Overall, the co-regulated phosphoproteins identified with Tau phosphosites in this study highlight a potential role for Tau in cancer development and progression. The observed phosphorylation patterns indicate that Tau may functionally relate to DNA damage response and repair mechanisms. This interaction suggests that Tau-associated signalling networks may be used by cancer cells to maintain genomic integrity, thereby highlighting Tau as a context-dependent regulator of oncogenic cellular processes. However, further experimental validations are required to substantiate these data-driven findings and to evaluate their potential translational relevance.

## 5 Conclusion

This study reveals Tau phosphorylation by integrating human cellular phosphoproteomics datasets across diverse experimental and disease contexts. Through systematic data curation and frequency-based evaluation, four predominant Tau phosphosites, S519, S713, S717, and S721, were consistently identified across profiling and differential phosphoproteomics studies. Co-regulation analysis exhibited an extensive network of protein phosphosites that were positively and negatively associated with the predominant Tau phosphosites, highlighting strong site-specific phosphorylation regulation. Phosphosite S519 exhibited a significant regulatory node, showing a pronounced bias toward positive co-regulation and broad connectivity with phosphosites involved in key cellular processes. Functional enrichment of co-regulated proteins related to Tau phosphorylation shows significant association with biological processes such as DNA damage response, cell cycle regulation, apoptosis, and cell growth, suggesting its roles beyond canonical neuronal functions. Protein-protein interaction data exhibited that Tau participates in both binary and complex interactions, supporting its involvement in broader signalling networks. Additionally, upstream kinase analysis revealed cancer signalling-related kinases such as CDKs, PRKCA and GSK3β, as potential regulators of Tau phosphorylation. Overall, this work creates a phosphoproteomic framework for Tau phosphorylation at specific phosphosites. By connecting predominant Tau phosphosites to co-regulatory networks, upstream kinases, and biological processes, the study offers a resource for future research into phosphosite-specific Tau regulation and its interaction with complex cellular signalling systems.

## 6 Limitations of the study

This study has several inherent limitations. It is based on the integration of multiple publicly available mass spectrometry-derived phosphoproteomics datasets and does not include experimental validation under controlled conditions. As a result, the identified Tau phosphosite patterns, co-regulation relationships, and inferred functional associations are derived from computational analyses and remain predictive, requiring targeted experimental validation to establish their biological and functional significance, particularly in cancer-related contexts.

The datasets analyzed originate from diverse experimental platforms, sample types, and analytical workflows, introducing variability in sample preparation, detection sensitivity, and data quality. Although stringent inclusion criteria and multi-layered filtering strategies were applied to improve robustness, residual technical variability, batch effects, and potential biases such as unequal representation of experimental conditions cannot be fully excluded.

The phosphosite-centric analytical framework depends on the detectability and recurrence of phosphosites across datasets, which may bias the analysis toward frequently observed sites while underrepresenting low-abundance or context-specific phosphorylation events. In addition, limitations inherent to bottom-up proteomics restrict the ability to resolve proteoform-specific phosphorylation patterns, thereby limiting isoform level interpretation.

Overall, the lack of experimental validation limits the ability to draw casual conclusions from the identified associations. However, this study offers a comprehensive, data-driven framework that provides a basis for generating testable hypotheses. Future experimental approaches, such as site-directed mutagenesis, phospho-specific antibody-based studies, kinase and phosphatase assays, and genome editing strategies including CRIPSR/Cas9-mediated phosphosite modification, will be important to confirm the functional relevance of the identified phosphosites. Applying these approaches in cell lines, patient-derived samples, and disease relevant models will help to establish mechanistic links between the predicted phosphorylation networks and observed disease phenotypes.

## Data Availability

The original contributions presented in the study are included in the article/Supplementary Material, further inquiries can be directed to the corresponding authors.
